# Ablation, percolation, and afterdepolarization

**DOI:** 10.1016/j.bpj.2026.05.016

**Published:** 2026-05-14

**Authors:** Daisuke Sato, Donald M. Bers

**Affiliations:** 1 Department of Pharmacology, University of California, Davis, Davis, CA, USA

## Abstract

Ablation is an effective treatment for cardiac arrhythmias, and its primary goal is to remove excitability from cells to break pathological reentrant circuits or focal activities and thereby prevent recurrent arrhythmias. Ablation prevents excitation by destroying cells in targeted regions. However, if ablation does not eliminate sufficient cells, waves of action potentials (APs) may continue to propagate, and ablation may fail to suppress arrhythmias. In this study, we develop a theoretical approach that predicts the minimum number of ablated cells to break reentrant circuits and suppress the initiation and propagation of focal activities. Ablation creates a mixture of excitable and nonexcitable cells. As the fraction of excitable cells increases, the probability of AP wave propagation increases, since an AP can only propagate when an excitable cell excites another excitable cell. When this fraction exceeds the “percolation threshold,” as defined in percolation theory, AP waves always propagate successfully, regardless of the size of the ablated area. Therefore, for reentrant arrhythmias, the fraction of excitable cells in the ablation area must be below the percolation threshold to block AP waves. For focal arrhythmias, delayed afterdepolarizations (DADs) can trigger premature ventricular contractions (PVCs) when DADs overcome the source-sink mismatch. Ablation of excitable cells also reduces the functional electrical sink, making it easier to initiate triggered activities. However, if the fraction of excitable cells in the ablation area is below the percolation threshold, triggered activities also cannot propagate. On the other hand, when the fraction of excitable cells is high, even though AP waves can propagate more easily, the initiation of PVCs becomes more difficult due to the source-sink mismatch. The propensity for PVCs reaches its maximum at the percolation threshold. These scenarios can also apply to other pathological conditions, such as myocardial ischemia and infarction, where excitable myocytes are interspersed with nonexcitable tissue.

## Significance

Cardiac ablation is a common procedure used to treat arrhythmias by destroying small areas of heart tissue that trigger irregular heartbeats. However, the optimal amount of tissue to ablate remains unclear. Too little ablation may not effectively prevent arrhythmias, while too much can paradoxically increase arrhythmia risk and impair heart function. This study uses a theoretical model to predict the minimum ablation required to suppress arrhythmias based on percolation theory and the source-sink relationship. The model reveals that arrhythmia risk is highest when the fraction of excitable cells in the ablated area is near the percolation threshold. These findings provide a new framework for understanding how ablation affects arrhythmogenesis and could help optimize ablation strategies to improve efficacy and safety.

## Introduction

Ablation is a clinically effective treatment for both reentrant and focal arrhythmias.^1^^,^^2^^,^^3^ For reentrant arrhythmias, ablation disrupts part of the reentrant circuit, while for focal arrhythmias, it directly eliminates the excitatory focus. In both cases, ablation destroys cells and renders a portion of cardiac tissue nonexcitable. However, excessive ablation can paradoxically promote arrhythmias and impair cardiac contraction. Therefore, it is crucial to minimize the ablation of healthy cells. Conversely, insufficient ablation may fail to break reentrant loops or eliminate focal activities. Unfortunately, there is currently no established approach for preclinically determining the necessary and sufficient ablation volume for a given patient.

Cardiac myocytes are excitable cells. In tissue, myocytes are coupled electrotonically via gap junctions, and action potentials (APs) can propagate from cell to cell as nonlinear waves.^4^ Ablation creates a mixture of excitable and nonexcitable cells within the tissue. Percolation theory is a mathematical framework used to study the connectivity of networks with randomly distributed elements. Ablated tissue can be mathematically described by percolation theory.^5^^,^^6^ For example, in the case of 1D tissue, if one cell in the 1D cable fails to be excited, AP propagation ceases. However, in 2D or 3D tissue, there are multiple paths that can bypass nonexcitable cells, allowing APs to continue propagating as long as the excitable paths remain connected. The key parameter is the fraction of excitable cells, *p*. Percolation theory predicts the existence of a sharp critical value, the percolation threshold *p*_*c*_, such that if *p* > *p*_*c*_, a connected path of excitable cells spans the tissue, whereas for *p* < *p*_*c*_, only isolated clusters exist.^7^^,^^8^ Previous studies have shown that fibrosis can also be described using percolation theory. Fibrosis not only creates passive conduction blocks but also forms long conduction pathways that effectively increase the tissue size capable of supporting sustained reentry.^9^^,^^10^ Moreover, as structural heterogeneity can initiate reentry,^11^ fibrosis can initiate micro-reentry, which, upon exiting, appears as focal ectopic activity.^12^

Another theoretical concept relevant to arrhythmias in cardiac tissue is the source-sink relationship, which describes the balance between the electric current provided by an excitatory source (e.g., a group of cells firing an AP) and the current required to excite the surrounding, resting tissue (the sink). This concept has been used to predict the likelihood of arrhythmias arising from premature ventricular contractions (PVCs), often initiated by delayed afterdepolarizations (DADs).^13^ When a cell is excited in tissue, the cell provides a source current to neighboring cells through gap junctions, while these neighboring cells act as a current sink. For AP wave propagation, the source current must overcome the electrical sink so that the neighboring cells can be depolarized above their excitation threshold.^14^^,^^15^^,^^16^^,^^17^^,^^18^ DADs are thought to be caused by spontaneous Ca^2+^ releases from the sarcoplasmic reticulum (SR) at the cellular level.^19^^,^^20^^,^^21^ These spontaneous Ca^2+^ releases increase intracellular Ca^2+^ and promote transient inward currents mostly through the Na^+^/Ca^2+^ exchanger (NCX), which depolarizes the membrane potential. Under healthy conditions, the spontaneous Ca^2+^ releases rarely occur. Even if they occur, a single DAD cannot initiate a PVC because the source current provided by the DAD is much smaller than the current by normal AP, and the neighboring cells quickly absorb the source current. This source-sink mismatch protects the heart from the occurrence of PVCs. In fact, previous studies^16^^,^^18^ showed that an isolated DAD from a single cell cannot initiate a PVC in tissue due to the source-sink mismatch. There are two ways to overcome the source-sink mismatch and promote the triggering of PVCs. One way is to increase the size of the source by increasing the number of cells generating a stimulus. The other way is to decrease the size of the sink by reducing the number of surrounding excitable cells. Although ablation prevents AP wave propagation, it simultaneously reduces the size of the sink, which could paradoxically promote arrhythmias under certain conditions.

In this paper, using percolation theory and the concept of source-sink relationship, we show that the propensity for PVCs to occur reaches a maximum when the fraction of excitable cells is near the percolation threshold. These principles allow us to predict the minimal ablation volume required to effectively suppress arrhythmias while minimizing damage to healthy tissue.

## Materials and methods

### Cellular and tissue models

We used a physiologically detailed rabbit ventricular AP model developed by Mahajan et al*.*^22^ This model incorporates detailed ionic currents, membrane voltage, and intracellular Ca^2+^ cycling. The governing equation for the membrane potential (*V*) is

(1) $$\frac{dV}{dt}=-\frac{1}{C_{m}}\left(I_{Na}+I_{CaL}+I_{Kr}+I_{Ks}+I_{K1}+I_{to,s}+I_{to,f}+I_{NaCa}+I_{NaK}\right)\text{,}$$

where *V* is the membrane potential, *C*_*m*_ = 1 μF/cm^2^ is the membrane capacitance, I_Na_ is the fast sodium (Na^+^) current, I_CaL_ is the L-type Ca^2+^ current, and I_Kr_ and I_Ks_ are the rapid and slow delayed rectifier K^+^ currents, respectively. I_K1_ is the inward rectifier K^+^ current, I_to,s_ and I_to,f_ are the slow and fast components of the transient outward K^+^ current, I_NaCa_ is NCX current, and I_NaK_ is the Na^+^-K^+^ pump current. All model equations and parameters are described in detail in Mahajan et al.^22^ While the results presented in this paper were obtained using this model, qualitatively similar results were also obtained using the Luo-Rudy phase 1 (LR1) and Echebarria-Karma models.^23^^,^^24^

Cardiac myocytes are electrotonically coupled via gap junctions in tissue. The AP propagation in the tissue is governed by the reaction-diffusion equation:

(2) $$C_{m}\frac{\partial V}{\partial t}=-I_{\text{ion}}+\sum_{k=1}^{n}G_{gap}^{k}\left(V^{k}-V\right)\text{,}$$

where *I*_*ion*_ is the total ionic current shown above (Equation 1), and $G_{gap}^{k}$ is the gap junction conductance between a cell and its *k*-th neighbor. The AP duration (APD) is approximately 200 ms, and the conduction velocity (CV) in the absence of obstacles is approximately 0.57 m/s. Thus, APD × CV ≈ 114 mm.

### Simulation protocols

**Figure 1:**
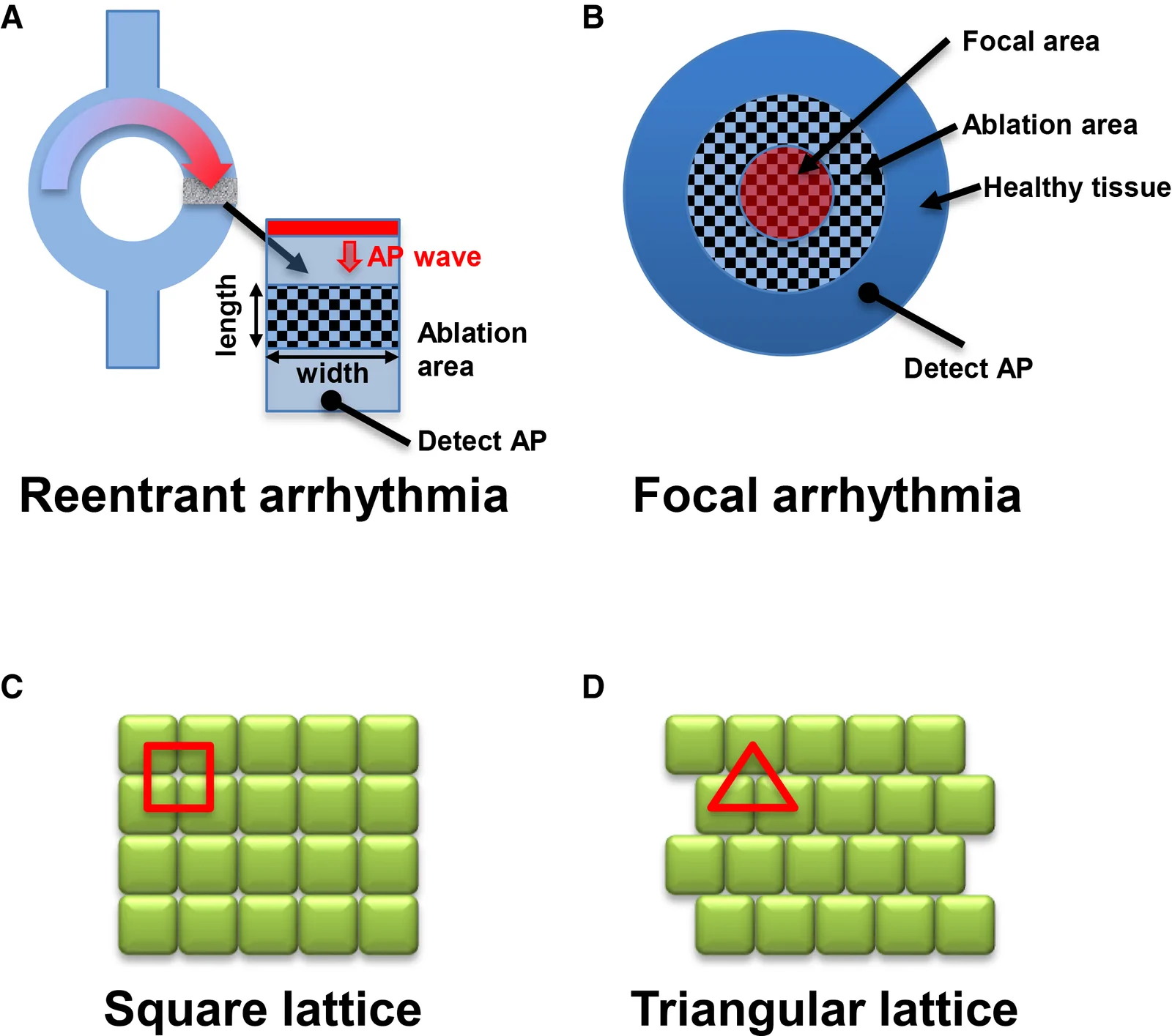
Simulation configurations for reentrant and focal arrhythmias. (A) Schematic diagram of the simulations of reentrant arrhythmia and (B) focal arrhythmia. The cell-to-cell network connectivity is modeled using either (C) a square lattice, where each cell has four neighboring cells, or (D) a triangular lattice, where each cell has six neighboring cells.

We modeled ablation in 2D tissue as shown in Figure 1. To investigate the relationship between the ablation area and the prevention of the AP wave propagation for reentrant arrhythmia, we varied the length of the ablation area and the fraction of excitable cells (living cells) (Figure 1A). We mixed excitable and nonexcitable cells uniformly at random. While in actual cardiac tissue cells often cluster into excitable or nonexcitable regions, this random mixing approximation is a reasonable starting point for the theoretical model as it allows us to explore the fundamental relationship between the proportion of excitable and nonexcitable cells and the probability of wave propagation and PVCs.

For focal arrhythmia, we varied the number of cells generating DADs and the fraction of excitable cells to investigate ablation of focal activities (Figure 1B). We used the formula by Xie et al.^18^ for the formation of DADs. Specifically, the spontaneous SR Ca^2+^ release flux was governed by

(3) $$J_{spon}=G_{spon}\times g_{1}\times g_{2}\times \left(\beta c_{j}-c_{s}\right)\text{,}$$

where *G*_*spon*_ = 0.0674 ms^−1^ is the maximum conductance, *β* is the submembrane space/SR volume ratio, and *c*_*j*_ and *c*_*s*_ are the Ca^2+^ concentrations in the junctional SR and submembrane space, respectively. The terms *g*_*1*_ and *g*_*2*_ are time-dependent functions defined as

(4) $$g_{1}=\frac{1}{1+\exp\left(-\frac{t-t_{0}}{\tau_{1}}\right)}\text{,}$$

(5) $$g_{2}=\frac{1}{1+\exp\left(\frac{t-t_{0}}{\tau_{2}}\right)}\text{,}$$

with t_0_ = 425 ms, τ_1_ = 10 ms, and τ_2_ = 30 ms. This Ca^2+^ released from the SR activates the transient inward current due to NCX that generates a DAD.

We also investigated how cell-to-cell connections affect the propagation and initiation of PVCs using two types of lattices; a square lattice (where one cell is surrounded by four cells) (Figure 1C) and a triangular lattice (where one cell is surrounded by six cells, which better reflects the geometry of actual cardiac tissue) (Figure 1D). For 3D tissue, we used a cubic lattice, where each cell is surrounded by six neighbors. Cells are coupled via gap junctions, as described by Equation 2, representing the site connections in percolation theory.

We first assumed that excitable cells are myocytes and nonexcitable cells are insulators like collagen. Subsequently, we considered more realistic tissue conditions to explore how fibroblasts affect AP propagation and PVC formation.

## Results

### Reentrant arrhythmia

**Figure 2:**
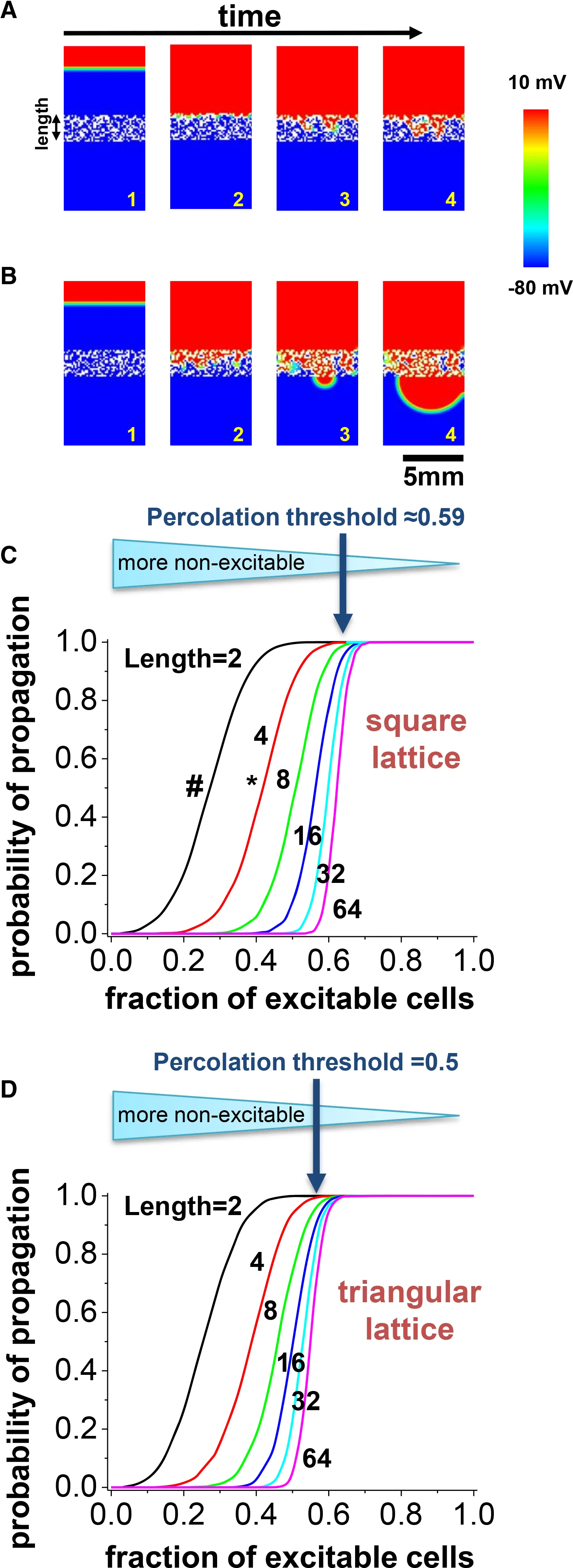
Effect of fraction of excitable cells on wave propagation. (A) Representative simulation where ablation successfully blocks AP wave propagation. White pixels indicate nonexcitable cells. (B) Representative simulation where ablation fails to block AP wave propagation. (C) The probability of AP wave propagation plotted against the fraction of excitable cells for varying lengths of the ablation area in a square lattice and (D) a triangular lattice. The width of the ablation area is fixed at 50 cells. Each data point represents the mean probability calculated over multiple simulation trials. The number of trials for each point was adjusted to ensure the standard error of the mean (SEM) was less than 0.01.

First, we investigated how many cells need to be ablated to prevent AP wave propagation. The probability that an AP wave propagates from one side of the ablation area to the other depends on its width and length. In this simulation, the width was fixed at 50 cells (0.75 cm). We varied the fraction of excitable cells, defined as

p=(thenumberofexcitablecells)/(thetotalnumberofcells).

(For example, if *p* is zero, all cells are nonexcitable. If *p* is 0.5, 50% of the cells are excitable, and the remaining cells are nonexcitable.) The excitable and nonexcitable cells were randomly distributed. If ablation renders all targeted cells nonexcitable (*p* = 0), the ablation area will always block AP wave propagation, regardless of its length. However, if some cells remain excitable and the length of the ablation area is short, the AP wave may penetrate the ablation area. This outcome depends on the fraction of excitable cells. Figure 2A demonstrates a case where the AP wave propagation is blocked by the ablation area (Figure 2A, Video S1), and Figure 2B, with fewer nonexcitable (white) cells, demonstrates a case where the AP wave propagates through the ablation area (Figure 2B, Video S2). If the length of the ablation area is 2 cells, 50% of AP waves successfully propagate when only 25% of the cells are excitable (indicated by **#**). When the length of the ablation area is increased to 4 cells, 50% of AP waves propagate when 50% of the cells are excitable (indicated by **^∗^**). As the length of the ablation area increases, a higher proportion of excitable cells is required for successful AP wave propagation through the ablation area (Figure 2C). However, once this fraction reaches a critical value, known as the percolation threshold (*p*_c_ **≈** 0.59 for a square lattice), the AP wave always propagates successfully through the ablation area, regardless of the length. We note that the threshold differs slightly from the theoretical value in our simulation due to the finite width of the simulated tissue, whereas theoretical values are calculated for an infinite space.

Video S1. Video file for Figure 2A

Video S2. Video file for Figure 2B

Our simulations suggest that if an ablation procedure leaves more than 59% of the cells excitable, it will not effectively block AP wave propagation. These results are when tissue is assumed to be a square lattice. In a tissue with a triangular lattice, where each cell is electrotonically connected to six neighboring cells, the percolation threshold is reduced to 0.5. As a result, the AP wave can penetrate more easily and propagate through the ablation area (Figure 2D).

### Focal arrhythmia

While the primary goal of ablation is to create nonconductive/nonexcitable tissue, this process of destroying cells also alters the source-sink dynamics of the remaining tissue. This may paradoxically promote arrhythmias from focal sources like afterdepolarizations. Afterdepolarizations typically have a smaller voltage amplitude compared with a normal AP. To initiate a PVC, a sufficient number of afterdepolarizations must occur in near synchrony to overcome the source-sink mismatch.^16^^,^^18^ When ablation destroys excitable cells, it is equivalent to reducing the functional sink. As a result, ablation might paradoxically increase the likelihood of PVC formation and subsequent arrhythmias.

To investigate this possibility, we simulated a focal source of DADs within the partially ablated tissue (Figure 1B). We applied a small transient inward current (*I*_ti_) due to the NCX, triggered by spontaneous Ca^2+^ releases (Equation 3) to 150, 200, 300, and 500 cells to mimic DADs (the details of the DAD protocol are described in Xie et al.^18^). A smaller *p* corresponds to a lower density of excitable cells and, consequently, a higher number of nonexcitable cells within the same area. In this study, we adjusted the radius of the focal area (Figure 1B) to keep the total number of DAD-generating cells constant.

**Figure 3:**
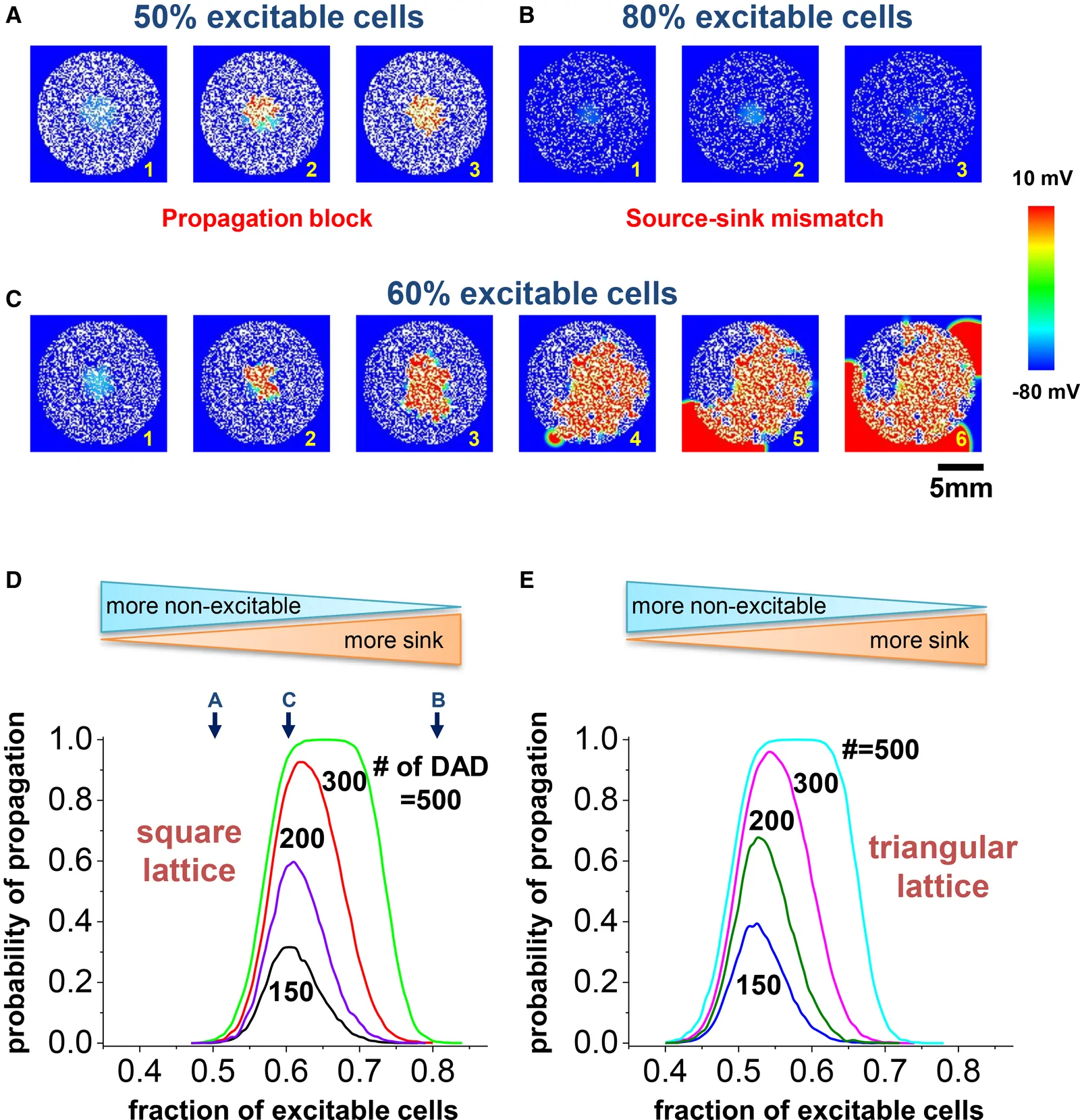
The propensity for focal arrhythmia peaks at the percolation threshold. (A) Triggered AP wave propagation is blocked when the fraction of excitable cells is below the percolation threshold. (B) Triggered activity fails to initiate due to source-sink mismatch when the fraction of excitable cells is too large. (C) Triggered activity successfully initiates and propagates when the fraction of excitable cells is near the percolation threshold. (D) The probability of successful AP wave propagation from a focal source versus the fraction of excitable cells in a square lattice and (E) a triangular lattice. The SEM for all data points is less than 0.01.

If *p* is too small (i.e., below the percolation threshold), any AP waves initiated by DADs will be blocked by the high density of surrounding nonexcitable cells (Figure 3A, Video S3) as the tissue lacks a connected pathway for propagation. Until *p* reaches *p*_*c*_ (*p* < *p*_*c*_), the probability of propagation follows the predictions of the percolation theory as described for Figure 2. Figure 3C (Video S5) shows an example where, with 60% excitable cells (*p* = 0.6, just above *p*_*c*_), a PVC is successfully initiated by DADs and propagates outward. As the fraction of excitable cells increases further (*p* > *p*_*c*_), the sink effect of the surrounding excitable cells becomes dominant, suppressing DADs initiating PVCs (Figure 3B, Video S4), thus decreasing the probability of PVCs. The probability of successful propagation is maximal at the percolation threshold (*p*_*c*_) in both square (Figure 3D) and triangular lattices (Figure 3E).

Video S3. Video file for Figure 3A

Video S4. Video file for Figure 3B

Video S5. Video file for Figure 3C

### Realistic 3D ablation model

**Figure 4:**
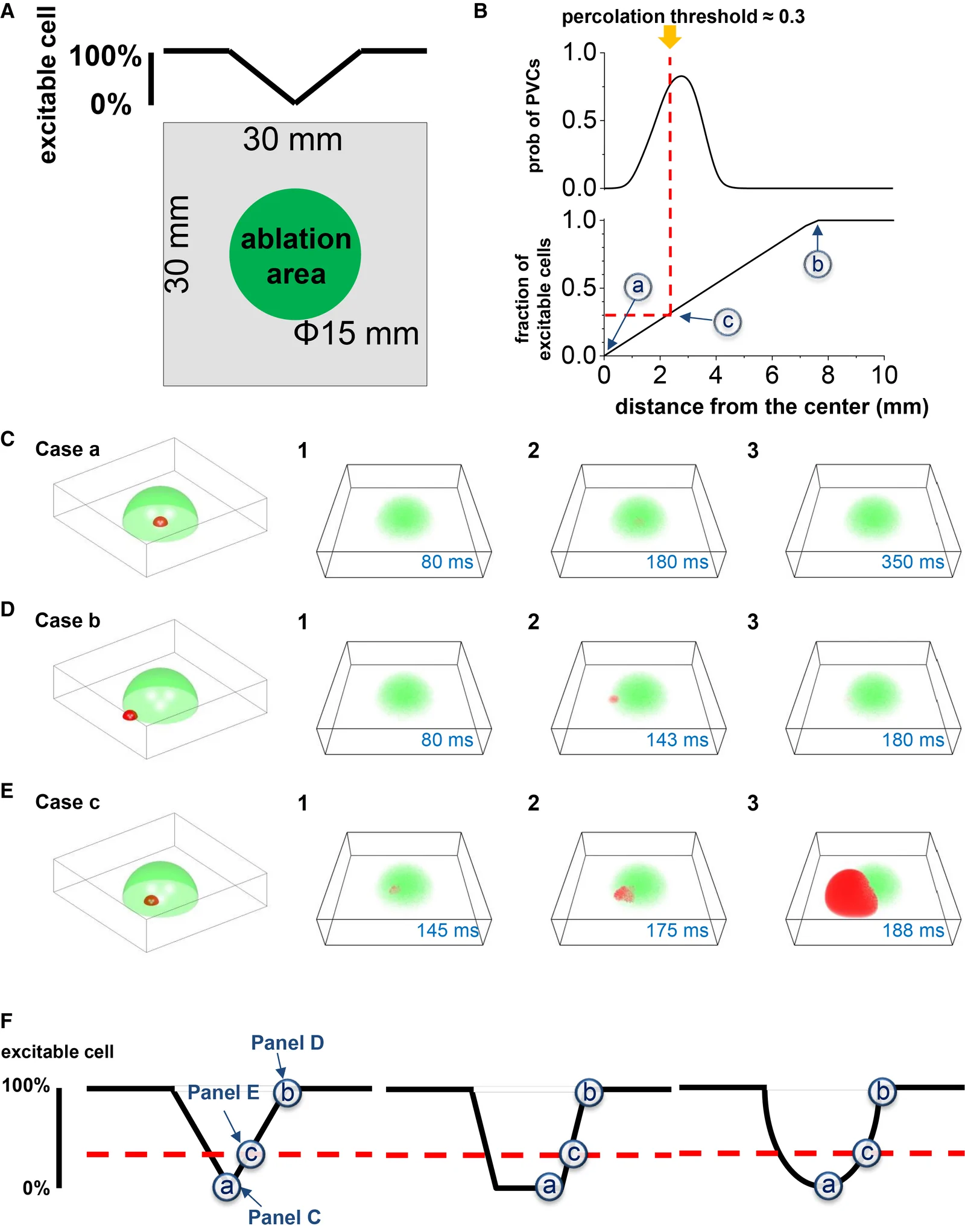
The propensity for PVCs becomes the maximum near the percolation threshold in a 3D ventricular ablation model. (A) Schematic diagram of the 3D ablation model. The tissue dimensions are 30 × 30 × 7.5 mm. The fraction of excitable cells increases linearly as a function of distance from the center of the ablation zone. (B) Top: probability of PVC propagation versus the distance between the center of the DAD area and the center of the ablation. Bottom: the fraction of excitable cells versus the distance from the center of the ablation. When the distance is approximately 2.25 mm, the fraction matches the percolation threshold (∼0.31). The points labeled a, b, and c correspond to the representative cases shown in (C), (D), and (E). (C) Case a: PVCs cannot propagate when the fraction of excitable cells is significantly lower than the percolation threshold. The green hemisphere indicates the ablation volume. The red hemisphere indicates the DAD-generating cells. Snapshots show wave propagation at the indicated simulation times. (D) Case b: DADs cannot overcome the source-sink mismatch to initiate a PVC when surrounded by excitable cells. (E) Case c: a PVC is successfully initiated and propagates when DADs occur in tissue where the fraction of excitable cells is near the percolation threshold. (F) In various scenarios (here we show three scenarios), (Case a) PVCs cannot propagate, (Case b) DADs cannot initiate a PVC, and (Case c) PVCs have the highest propensity to be initiated and propagate.

We next considered a more realistic scenario in a computational 3D reconstruction of a ventricular wall (30 × 30 × 7.5 mm). During cardiac ablation, cells are ablated especially near the tip of the catheter. We assumed that the ablation region (green hemisphere) has a diameter of 15 mm, with the number of ablated cells decreasing linearly as the distance from the catheter tip increases (Figure 4A). Specifically, the fraction of excitable cells (p) equals 0 at the center and linearly increases to 1 at a distance of 7.5 mm from the center.

We assumed that DADs occur in excitable cells (nonablated cells) within 1.5 mm radius (red hemispheres in Figures 4C, 4D, and 4E), which corresponds to the focal area in Figure 1B. We varied the location of these DAD-generating cells (focal area) from the center (Figure 4C) of the ablation toward the circumference (Figure 4E). PVCs occurred most frequently when DADs originated in tissues where the fraction of excitable cells was close to the percolation threshold for a 3D cubic lattice (*p*_c_ **≈** 0.31) (Figures 4B and 4E, case c in Figure 4F).

Our simulations predict that if DADs occur close to the center of the ablation region (where *p* < *p*_*c*_), the propagation of PVCs will be blocked by the high density of nonexcitable cells (Figure 4C, case a in Figure 4F, Video S6). On the other hand, if DADs occur far from the center of the ablation region (e.g., at its edge where *p* is much greater than *p*_*c*_), PVCs will not be initiated due to the source-sink mismatch (Figure 4D, case b in Figure 4F, Video S7). However, when DADs occur near the percolation threshold, they overcome the sink effect and initiate a PVC, and it can also propagate through the tissue (Figure 4E, case c in Figure 4F, Video S8). Cardiac tissue is heterogeneous, and the ablation of cells depends on the intrinsic properties of the tissue, such as thermal conductivity and susceptibility to ablation-related cell death. Nevertheless, the results shown here are applicable in various physiologically plausible scenarios (Figure 4F shows 3 example ablation geometries and the probability of successful propagation is maximal at c in all cases).

Video S6. Video file for Figure 4C

Video S7. Video file for Figure 4D

Video S8. Video file for Figure 4E

### Fibroblasts

**Figure 5:**
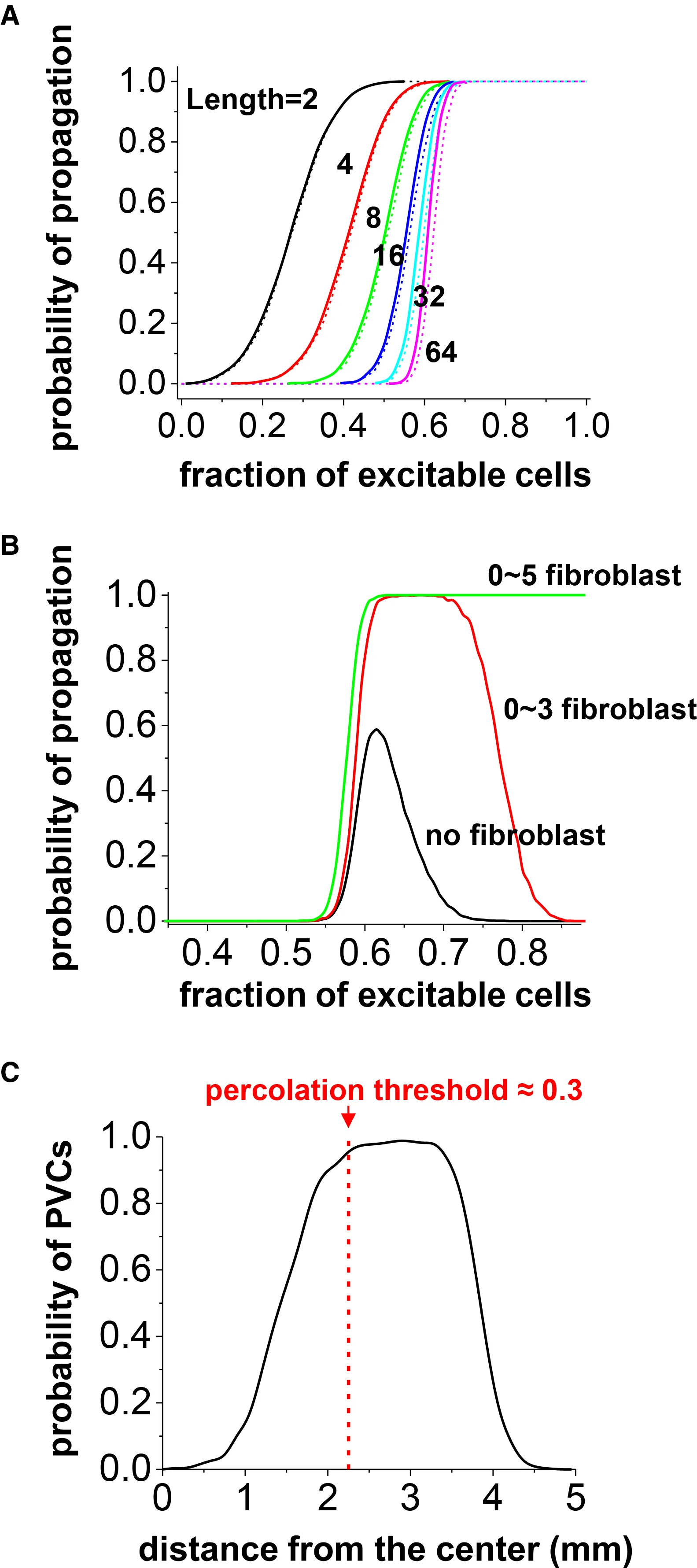
Impact of fibroblasts. Fibroblasts affect the source-sink relationship but not the threshold. (A) The probability of wave propagation remains largely unchanged in the presence of fibroblasts. Solid lines: 0 to 5 fibroblasts attached to each myocyte. Dashed lines: no fibroblasts (identical to Figure 2C). Simulations were performed on a square lattice. (B) Fibroblasts depolarize the resting membrane potential, thereby reducing the electrical sink. The probability of focal wave propagation versus the fraction of excitable cells. Black line: no fibroblasts. Red line: 0 to 3 fibroblasts attached to each myocyte. Green line: 0 to 5 fibroblasts attached to each myocyte. Simulations were performed on a square lattice. (C) The propensity for PVCs continues to reach its maximum near the percolation threshold in the 3D ventricular wall model, even with the addition of fibroblasts. The SEM for all data points is less than 0.01.

When ablation is applied, fibroblasts also appear in the healing tissue. Fibroblasts were modeled following Xie et al.^25^ and coupled to the myocytes. When 0 to 5 fibroblasts were randomly attached to each myocyte, the probabilities of successful propagation were almost the same as in the case without fibroblasts (Figure 5, solid lines versus dashed lines). However, because fibroblasts have a more depolarized resting membrane potential than myocytes, they slightly depolarize the resting membrane potential of the coupled myocytes, reducing the sink required for PVC propagation. As a result, PVCs can occur at a higher fraction of excitable cells compared with tissue without fibroblasts (Figure 5B, red and green curves). Nonetheless, the propensity for PVCs to occur -remained maximal at the percolation threshold in all cases. Lastly, we added fibroblasts to the 3D tissue simulated in Figure 4. The occurrences of PVCs became maximal near the percolation threshold (Figure 5C).

## Discussion

Ablation suppresses reentrant and focal arrhythmias by removing excitability from a part of the cardiac tissue. However, excitability is the central property of the cardiac cells that is required for contraction and maintenance of the pumping function of the heart. Therefore, reducing excitability by ablation must be carefully limited to preserve primary cardiac function. However, approximately 30% of ablation procedures fail clinically, requiring multiple procedures or alternative invasive methods.^26^ Currently, there is no reliable method to map the necessary and sufficient ablation area prior to the onset of the invasive procedure. It is critically important to develop methodologies that can identify the minimum number of ablated cells required to prevent the propagation of arrhythmic rhythms. In this study, we investigated the universal properties of ablated tissue, focusing on the relationship between ablation size, the percolation threshold, and the propensity for arrhythmia.

Our main finding is the nonmonotonic relationship between the density of excitable cells and the propensity for PVCs. Our results show that although an ablation procedure creates obstacles that can impede and ultimately block wave propagation, replacing excitable myocytes with nonexcitable elements reduces the electrical sink as well. This reduction facilitates arrhythmogenesis by making it easier for a small focal source, which would normally be suppressed by the sink, to successfully initiate a wave. Our study demonstrates that the interplay between these two competing effects, easier initiation versus harder propagation, is critically determined by the connectivity of the tissue. In the sub-critical regime (*p* < *p*_*c*_), even if a PVC is initiated, it cannot propagate. In the super-critical regime (*p* > *p*_*c*_), the tissue is well connected, but the large electrical sink suppresses the initiation of PVCs. The propensity for arrhythmia is therefore maximized at the percolation threshold (*p*_*c*_), where the sink is just small enough to be overcome, and the network is just connected enough to sustain propagation.

Therefore, cell-to-cell network connectivity is one of the critical factors that determines this nonmonotonic relationship, especially the peak. In this study, to investigate the effects of cell-to-cell network connectivity, we tested three types of lattices: a square lattice (2D), a triangular lattice (2D), and a cubic lattice (3D). Cardiac AP waves propagate when excited cells excite the neighboring cells. If the neighbors are nonexcitable, such as collagen or dysfunctional cells, the AP wave terminates at that point. The basis of ablation is to render excitable cells nonexcitable in order to block reentry or focal activities. Cardiac tissue can be formed as a 1D cable (e.g., Purkinje fibers), a 2D sheet (e.g., the thin wall of the atria and right ventricle), or a 3D slab (e.g., the thick wall of the left ventricle). If tissue is 1D, a single nonexcitable cell is sufficient to block propagation. However, if tissue is 2D or 3D, a single nonexcitable cell cannot block propagation entirely, as the AP wave can bypass it. The AP wave will propagate as long as there is a continuous path connecting one side of the tissue to the other. If ablation is perfect, meaning it completely eliminates all excitable cells in the targeted area, a thin layer of nonexcitable cells is sufficient. However, if ablation leaves a fraction of cells excitable, the ablated area may retain contiguous conductive pathways that connect one side of the tissue to the other. Our results demonstrate that when the number of excitable cells exceeds the percolation threshold, AP waves can readily propagate from one side to the other side, and the alignment of cells in the tissue affects AP wave propagation as predicted by percolation theory (Figures 2C, 2D, and 5A). Experimental measurements have shown that a single cell is typically surrounded by about 11 cells on average.^27^^,^^28^ In this case, the percolation threshold will be much lower (*p*_c_ **≈** 0.2 if each cell is surrounded by 12 cells) for the 3D tissue,^29^ and a small number of excitable cells is sufficient to maintain a reentrant circuit.

In addition to cell-to-cell network connectivity, we also varied the size of the ablation area by changing the length in Figure 1A and the size of the focal area in Figure 1B. Other factors such as electrophysiological properties can also affect the nonmonotonic relationship. For example, a reduction in sodium channel conductance or an increase in I_K1_ current raises the excitation threshold and thus requires more source current to excite. Similarly, reduced gap junction coupling reduces intercellular current, necessitating greater source current. On the other hand, a longer action potential duration provides more source current.

In this study, we first showed this nonmonotonic relationship between the density of excitable cells and the propensity for PVCs using cases where *p* is constant (Figures 3D, 3E, and 5B). In a realistic setting of tissue, *p* varies by location.^30^ In our model, we assumed *p* increases linearly from the center of the ablation area to the outer normal tissue. We found that the peripheral area where the fraction of excitable cells approaches the percolation threshold exhibits the maximum propensity for PVCs (Figures 4 and 5C). Importantly, our simulations across multiple geometric configurations (Figure 4F) demonstrate that this phenomenon is fundamentally geometry independent. These results also provide a mechanistic explanation for why PVCs are often observed at the border zone of myocardial infarction,^31^^,^^32^ independent of electrophysiological changes in infarcted tissue. Following a myocardial infarction, the heart heals into a heterogeneous tissue, not a uniform scar. A heterogeneous border zone forms, characterized by a complex intermingling of surviving excitable myocytes and nonexcitable fibrotic tissue. It has been recognized that this border zone, not the dense scar core or the healthy remote tissue, is the primary source of post-infarct ventricular arrhythmias.^31^^,^^32^ Our model provides a mechanistic basis for this observation. The dense scar core (*p* < *p*_*c*_) is too disconnected for a wave to propagate. In healthy tissue (*p* > *p*_*c*_), the source-sink mismatch is large, which suppresses initiation. Between these regimes, *p* becomes close to the percolation threshold (*p* ≈ *p*_*c*_) where arrhythmia risk is maximal. In this zone, there is enough fibrosis to significantly reduce the electrical sink, making it easier for a focal trigger to initiate a PVC. Simultaneously, there are still just enough surviving, interconnected myocytes to form a continuous pathway for that PVC to propagate out of the border zone and into the healthy ventricle, potentially triggering a life-threatening arrhythmia. This framework also applies directly to other conditions involving cardiac fibrosis, such as hypertrophic cardiomyopathy or age-related fibrosis, where fibrosis is often diffuse and patchy. Our model predicts that areas with an intermediate level of fibrosis would be the most likely to generate and sustain focal arrhythmias. Previous studies have shown that fibrosis can serve as a substrate for both sustained reentry^9^^,^^10^ and focal ectopic activity due to micro-reentry.^12^ Our study provides another mechanism that promotes arrhythmia, providing a more detailed view than simply stating “fibrosis is arrhythmogenic”; it suggests that the pattern and density of fibrosis are the critical determinants of arrhythmia risk.

Finally, in this study, we used the Mahajan et al. model,^22^ and the results were qualitatively consistent across different excitable models. In addition to the Mahajan et al. model, we also tested the Luo-Rudy phase 1 model^23^ and the Echebarria-Karma model^24^ and obtained similar results (data not shown).

### Conclusion

In this study, we have shown that (1) the propagation of AP waves in an ablation area obeys percolation theory. Above the percolation threshold, AP waves always propagate, regardless of the size of the ablated area. This implies that for ablation to be effective against reentry, it must reduce the density of excitable cells below this critical threshold. (2) Ablation reduces the electrical sink effect, which can paradoxically promote the initiation of PVCs. (3) The propensity for PVCs is maximal near the percolation threshold, where the sink is small enough to be overcome by a focal source, but the network of excitable cells is still connected enough to support propagation. These conclusions are independent of the AP model used or species. Our findings provide a theoretical foundation for both the prevention and the potential induction of arrhythmias through ablation.

## Data and code availability

The authors confirm that the data supporting the findings of this study are available within the article. The simulation code is available on GitHub or upon request.

## Acknowledgments

This work was supported by 10.13039/100000002NIH grants (P01-HL141084 and R01HL142282 to D.M.B., R01HL149349 to D.S. and D.M.B.) amd American Heart Association grant (26RIRA1614813 to D.S.).

## Author contributions

D.S. performed mathematical modeling and computer simulations. D.S. processed and analyzed the simulation data. D.S. and D.M.B. interpreted the data and wrote the manuscript. All authors approved the final version of the manuscript.

## Declaration of interests

The authors declare no conflict of interests.
